# Predictive Factors for Early Immune Recovery in NHL Patients after Autologous Transplantation: A Multicenter Prospective Study

**DOI:** 10.3390/cancers16142550

**Published:** 2024-07-16

**Authors:** Anu Partanen, Antti Turunen, Outi Kuittinen, Hanne Kuitunen, Pentti Mäntymaa, Ville Varmavuo, Esa Jantunen

**Affiliations:** 1 Department of Medicine, Kuopio University Hospital, 70290 Kuopio, Finland; antti.s.turunen@pshyvinvointialue.fi (A.T.); esa.jantunen@pshyvinvointialue.fi (E.J.); 2 Institute of Clinical Medicine, University of Eastern Finland, 70211 Kuopio, Finland; outi.kuittinen@uef.fi; 3 Department of Oncology, Kuopio University Hospital, 70290 Kuopio, Finland; 4 Cancer Center, Oulu University Hospital, 90220 Oulu, Finland; hanne.kuitunen@ppshp.fi; 5 Eastern Finland Laboratory Centre, 70211 Kuopio, Finland; pentti.mantymaa@islab.fi; 6 Department of Medicine, Kymenlaakso Central Hospital, 48210 Kotka, Finland; ville.varmavuo@iki.fi

**Keywords:** early immune recovery, autologous hematopoietic stem cell transplantation, non-Hodgkin lymphoma, graft cellular composition, outcome

## Abstract

**Simple Summary:**

Early lymphocyte recovery has been associated with a better outcome after autologous hematopoietic stem cell transplantation in many retrospective studies. Scarce prospective data exist on the detailed factors that correlate with early immune recovery as manifested by the absolute lymphocyte count at day +15 after transplantation and the influence of early immune recovery on outcome in patients with non-Hodgkin lymphoma. This prospective multicenter study aimed to clarify factors linked with early lymphocyte recovery after autologous stem cell transplantation among 178 patients with non-Hodgkin lymphoma. We found that faster early immune recovery was strongly associated with better PFS. Specific cut-offs of mobilization capacity as manifested by peak blood CD34^+^ cell count as well as CD3^+^CD4^+^ T cells, CD3^+^CD8^+^ T cells, and NK cells in the infused graft seemed to predict early immune recovery, which is associated with a better outcome in NHL patients after transplantation.

**Abstract:**

Early lymphocyte recovery as manifested by an absolute lymphocyte count at d+15 (ALC-15) ≥ 0.5 × 10^9^/L after autologous hematopoietic stem cell transplantation (AHCT) has been associated with a better outcome. This prospective multicenter study aimed to clarify factors associated with ALC-15 ≥ 0.5 × 10^9^/L after AHCT among 178 patients with non-Hodgkin lymphoma. The mobilization capacity, as manifested by peak blood CD34^+^ cell numbers > 45 × 10^6^/L correlated with higher ALC-15 levels (*p* = 0.020). In addition, the amount of CD3^+^CD4^+^ T cells > 31.8 × 10^6^/kg in the infused graft predicted ALC-15 ≥ 0.5 × 10^9^/L (*p* < 0.001). Also, the number of infused graft CD3^+^CD8^+^ T cells > 28.8 × 10^6^/kg (*p* = 0.017) and NK cells > 4.4 × 10^6^/kg was linked with higher ALC-15 (*p* < 0.001). The two-year progression-free survival after AHCT was significantly better in patients with ALC-15 ≥ 0.5 × 10^9^/L (74 vs. 57%, *p* = 0.027). The five-year OS in patients with higher ALC-15 was 78% vs. 60% in those with lower ALC-15 (*p* = 0.136). To conclude, the mobilization capacity of CD34^+^ cells and detailed measures of graft cellular content mark prognostic tools that predict ALC-15 ≥ 0.5 × 10^9^/L, which is associated with a better outcome in NHL patients after AHCT.

## 1. Introduction

Autologous hematopoietic stem cell transplantation (AHCT) is an option for cure or prolonged survival for a proportion of patients with non-Hodgkin lymphoma (NHL). The optimal graft content needed for AHCT to support both early and long-term hematological and immunological recovery as well as long-term outcomes is unclear.

Early lymphocyte recovery, defined as an absolute lymphocyte count at d+15 (ALC-15) ≥ 0.5 × 10^9^/L after AHCT, has been associated especially with prolonged progression-free survival (PFS) [1] but also with an overall survival (OS) benefit [2,3] in NHL patients. Early immune recovery is of importance due to two factors: it may protect against early infections, and it may also play a defensive role against the underlying lymphoma [4,5,6]. According to previous studies with a limited number of patients, the number of graft NK cells correlates with ALC-15 [7,8]. Plerixafor use, which enhances the mobilization of various lymphatic cells, including NK cells [9,10,11], has been linked to ALC-15 ≥ 0.5 × 10^9^/L. In addition, the number of more primitive CD34^+^CD133^+^CD38^−^ cells in the infused grafts has been associated with early lymphocyte recovery [12]. 

Scarce prospective data exist on the detailed factors that correlate with early immune recovery and the influence of early immune recovery on outcome in patients with NHL. The prospective non-interventional graft and outcome in autologous stem cell transplantation (GOA) study focused on the influence of different mobilization methods on the graft cellular composition and correlations of graft cellular composition with hematologic and immune recovery as well as outcome after AHCT. The scope of the present study, as a part of the prospective GOA study, was to find out the factors predicting early immune recovery defined by ALC-15 ≥ 0.5 × 10^9^/L and the impact of early immune recovery on PFS and OS in NHL patients after AHCT.

## 2. Material and Methods

### 2.1. Patients and Methods

Between May 2012 and December 2018, a total of 178 adult patients with NHL undergoing AHCT at three university hospitals in Finland (Kuopio, Oulu, and Tampere) participated in the non-interventional multicenter GOA study. The patient characteristics and demographics are presented in Table 1.

### 2.2. Mobilization and Collection of Blood Grafts

All the patients received chemotherapy and a granulocyte colony-stimulating factor (G-CSF) chosen by a treating physician to mobilize blood grafts. A total of 62 (35%) patients needed plerixafor rescue (median 2 injections, range 1–4). Except for the time frame before April 2013 at Kuopio University Hospital, where a COBE Spectra AutoPBSC apheresis machine (Lakewood, CO, USA) (n = 26) was used, Spectra Optia Apheresis systems (COBE Laboratories Inc., Lakewood, CO, USA) (n = 122 (KUH), n = 26 (OYS), n = 4 (TAYS)) were used in apheresis. The minimal collection goal was 2 × 10^6^/kg CD34^+^ cells, and analyses of CD34^+^ cell counts using the ISHAGE protocol [13] were performed from each apheresis bag after the collection. For later graft cellular analyses, two additional 0.5 mL tubes of each apheresis product were taken. After adding dimethylsulfoxide (final concentration of 10%), the samples were handled and preserved in the vaporing phase of liquid nitrogen, like the grafts.

### 2.3. The Graft Cellular Analysis

A single experienced flow cytometrist at the Department of Clinical Microbiology, University of Eastern Finland, later performed all flow cytometry analysis (FACSCanto, Becton Dickinson, Franklin Lakes, NJ, USA) of the thawed cryopreserved graft samples. An ISHAGE protocol with a single-platform method [13] was used to evaluate both the number of CD34^+^ cells and lymphocyte subclasses. Antibodies against the following cell surface markers were used: CD34, CD38, CD133, and CD45. All antibodies except CD133 (Miltenyi Biotec GmbH, Bergisch Gladbach, Germany) were delivered by Becton Dickinson. Viable CD34^+^ cells were distinguished by 7-aminoactinomycin (7-AAD) staining. To assess the absolute counts of T, B, and NK cells as well as the CD3^+^CD4^+^ and CD3^+^CD8^+^ subpopulations of T cells in the grafts, both CD3/CD8/CD45/CD4 and CD3/CD16^+^CD56/CD45/CD19 reagents (BD Multitest, Becton Dickinson) with tubes (BD Trucount, Becton Dickinson) were used.

### 2.4. High-Dose Therapy and Posttransplant Course

All participating patients with systemic lymphoma received either BEAM (carmustine 300 mg/m^2^ on day −6, etoposide 200 mg/m^2^ from day −5 to day −2, cytarabine 300 mg/m^2^ from day −5 to day −2 and melphalan 140 mg/m^2^ on day −2) (n = 152; 85%) or BEAC (carmustine 300 mg/m^2^ on day −7, etoposide 200 mg/m^2^ from day −6 to day −3, cytarabine 200 mg/m^2^ from day −6 to day −3 and cyclophosphamide 1500 mg/m^2^ from day −6 to day −3) (n = 7; 4%) as a high-dose therapy. Patients with primary central nervous system lymphoma (PCNSL) (n = 19) received carmustine 400 mg/m^2^ on day −6 and thiotepa 5 mg/kg ×2/day from day −5 to day −4. 

Neutrophil engraftment was defined as an absolute neutrophil count > 0.5 × 10^9^/L. Corresponding platelet (PLT) engraftment was defined as a platelet number > 20 × 10^9^/L without PLT transfusions for the previous three days. A cut-off point of ≥0.5 × 10^9^/L for absolute lymphocyte count at d + 15 after transplantation (ALC-15) was used as the definition for early immune recovery. To evaluate hematologic and immunologic recovery complete blood counts were obtained on day +15 and at 1, 3, 6, and 12 months after AHCT. The follow up was continued for at least 5 years after AHCT.

### 2.5. Statistical Analysis

All calculations and statistical analyses were performed with the statistical program package SPSS (IBM SPSS Statistics Version 29.0, Chicago, IL, USA). Continuous numerical variables were described using medians with ranges. Descriptive statistics for categorical variables were presented as frequencies and percentages. The missing values were missing randomly, and the results of the analysis represent the whole study population. Statistical analysis was performed with a regression model for binary variables to study associations between ALC-15 ≥ 0.5 × 10^9^/L and patient and mobilization characteristics as well as infused graft cellular components. Univariate and multivariate analyses were carried out. The results of those were presented using OR and 95% confidence intervals (CIs). The optimal cut-off points for cellular component of the infused graft correlating with ALC-15 ≥ 0.5 × 10^9^/L were defined by using ROC curves. Progression-free survival (PFS) and overall survival (OS) were analyzed by using the log-rank test and Kaplan-Meier’s method. Two-tailed *p* values < 0.05 were considered significant.

### 2.6. Ethics

The Research Ethics Committee of the North Savo Hospital District (13/2012) approved the GOA study protocol, and the amendment regarding inclusion of NHL patients transplanted in 2017–2018 was also later approved (2/2018). The study was conducted according to the Declaration of Helsinki. A written informed consent was obtained from all participating patients.

## 3. Results

### 3.1. CD34^+^ Cell Mobilization and Graft Cellular Analysis

The median number of aphereses was 2 (range 1–4), and altogether 50% of the patients needed only a single apheresis session to achieve adequate graft. The median number of CD34^+^ cells collected was 3.6 × 10^6^/kg (1.6–25.4). Four patients not reaching the collection target > 2 × 10^6^/kg CD34^+^ cells also proceeded to AHCT according to the clinician’s decision. The graft collection data are presented in Table 2.

A total of 129 patients had cellular analysis of thawed grafts available. The median number of infused viable graft CD34^+^ cells was 2.5 × 10^6^/kg (0.6–14.3) and more primitive CD34^+^CD133^+^CD38^−^ cells were 0.073 × 10^6^/kg (0.006–0.346), respectively. Regarding lymphocyte subgroups, the median number of cryopreserved CD3^+^ cells was 88.8 × 10^6^/kg (0.7–1022.5), CD3^+^CD4^+^ T-helper cells 44.2 × 10^6^/kg (0.5–365.3), cytotoxic CD3^+^CD8^+^ cells 34.3 × 10^6^/kg (0.4–644.9), and NK cells 6.1 × 10^6^/kg (0.05–56.9). Due to rituximab use in the great majority of the patients, the median number of CD19^+^ B-cells was low (0.0 × 10^6^/kg, range 0.0–100.2).

A significant correlation was detected between the number of apheresis sessions and the graft CD3^+^ cell count (*r* = 0.368, *p* < 0.001), CD3^+^CD4^+^ cell count (*r* = 0.268, *p* = 0.002), CD3^+^CD8^+^ cell count (*r* = 0.438, *p* < 0.001), and the number of NK cells (*r* = 0.395, *p* < 0.001). In patients with plerixafor use, only the amount of CD3^+^ cells significantly correlated with the number of aphereses (*r* = 0.391, *p* = 0.020), whereas in plerixafor-naïve patients both CD3^+^CD8^+^ cell number (*r* = 0.294, *p* = 0.004) and NK cell number (*r* = 0.218, *p* = 0.035) correlated with the number of aphereses. 

### 3.2. Predictive Factors for ALC-15

The median ALC-15 was 0.6 × 10^9^/L (range 0.04–4.2), and altogether 79 patients (43%) had ALC-15 ≥ 0.5 × 10^9^/L after AHCT. Lymphoma type, disease status at the time of transplantation, G-CSF type used, or need for plerixafor in the mobilization had no correlation with ALC-15 ≥ 0.5 × 10^9^/L. Instead, the number of days from the start of mobilization to AHCT was linked with ALC-15 ≥ 0.5 × 10^9^/L (*r* = −0.286, *p* < 0.001). The cut-off points for graft cellular content associating with ALC-15 ≥ 0.5 × 10^9^/L were 42.8 × 10^6^/kg for the number of CD3^+^ cells, 28.8 × 10^6^/kg for CD3^+^CD8^+^ cells, 31.8 × 10^6^/kg for CD3^+^CD4^+^ cells, and 0.09 × 10^6^/kg for primitive CD34^+^CD133^+^CD38^−^ cells analyzed by using ROC curves (Table 3). 

Mobilization capacity as manifested by peak blood CD34^+^ cell count > 45 × 10^9^/L predicted a higher ALC-15 level in a multivariate model (OR 5.157, CI 1.301–20.444, *p* = 0.020), and the cut-off point of peak blood CD34^+^ cell count > 45 × 10^9^/L was found in 82 (46%) of the patients. Neither the cut-offs for CD34^+^CD133^+^CD38^−^ cell count (*p* = 0.371) nor the CD34^+^ cell count with AAD (*p* = 0.897) of the infused grafts were correlated with ALC-15 ≥ 0.5 × 10^9^/L in a multivariate model, whereas a significant association with higher ALC-15 was detected in a univariate model for both parameters (*p* = 0.017 and *p* = 0.049). Despite the predictive role of infused graft CD3^+^ cell numbers > 42.8 × 10^6^/kg for ALC-15 ≥ 0.5 × 10^9^/L in the univariate analysis (OR 7.962, CI 2.891–21.925, *p* < 0.001), the association did not reach statistical significance in multivariate analysis (*p* = 0.065). The number of CD3^+^CD4^+^ T-helper cells > 31.8 × 10^6^/kg in the infused graft was strongly linked with ALC-15 ≥ 0.5 × 10^9^/L in the univariate model (OR 6.459, CI 2.713–15.375, *p* < 0.001). Also, graft CD3^+^CD8^+^ T-cell counts > 28.8 × 10^6^/kg correlated with higher ALC-15 in univariate analysis (OR 3.994, CI 1.788–8.919, *p* < 0.001). In addition, the number of NK cells > 4.4 × 10^6^/kg in the infused graft was linked with ALC-15 ≥ 0.5 × 10^9^/L (OR 2.635, CI 1.192–5.824, *p* = 0.017) in the univariate model, but this finding was not significant in the multivariate model (*p* = 0.750). (Table 4).

### 3.3. Survival Analysis

The median follow-up time was 49 months (range < 1–107) from AHCT. At the data cut-off point (28 March 2024), altogether 115 (66%) of the patients were progression-free, and 125 (70%) of the patients were alive. There was no statistically significant difference in the short-term PFS (<100 d) (13 vs. 8 relapses, *p* = 0.645) or OS (4 vs. 3 deaths, *p* = 0.769) according to the ALC-15 level. The great majority of the deceased patients (37/53; 70%) had experienced a lymphoma relapse, which was also a reason for death in 30 (58%) of those. 

**Figure 1:**
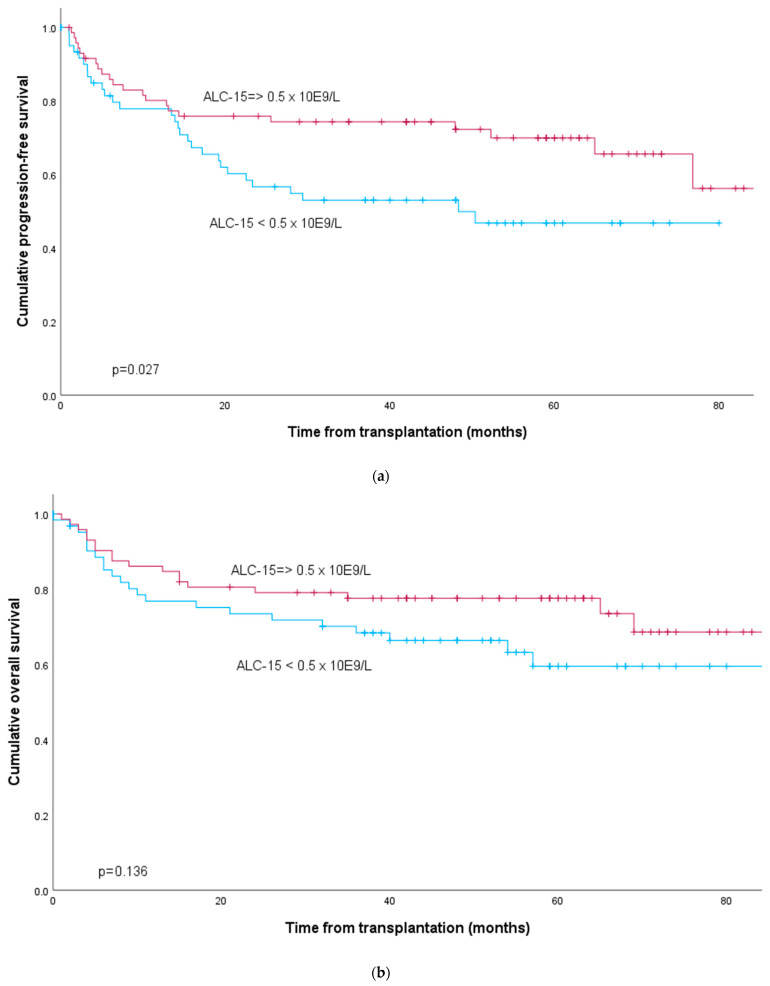
Progression-free survival (**a**) and overall survival (**b**) of 178 patients with NHL according to absolute lymphocyte counts at d + 15 (ALC-15) after AHCT.

The time to progression was significantly longer in the patients with ALC-15 ≥ 0.5 × 10^9^/L (48 months vs. not reached, *p* = 0.042). The two-year PFS after AHCT was 76% in those with ALC-15 ≥ 0.5 × 10^9^/L as compared to 57% in the patients with ALC-15 < 0.5 × 10^9^/L, and the same difference was also detected five years after transplantation (70 vs. 47%) (*p* = 0.027) (Figure 1). The five-year OS rate in patients with ALC-15 ≥ 0.5 was 78% vs. 60% in those with lower ALC-15, but this difference did not reach statistical significance (*p* = 0.136) (Figure 1).

## 4. Discussion

Most studies regarding the importance of early immune recovery in autologous settings have been retrospective [14] and are potentially confounded by publication bias. In the present prospective multicenter study, we found that the mobilization capacity as manifested by peak blood CD34^+^ cell count was associated with a higher ALC-15 level. In addition, the CD3^+^CD4^+^ T-helper cell count > 31.8 × 10^6^/kg in the infused graft strongly predicted ALC-15 ≥ 0.5 × 10^9^/L. Also, both infused CD3^+^CD8^+^ cell counts > 28.8 × 10^6^/kg and NK cell counts > 4.4 × 10^6^/kg correlated with ALC-15 ≥ 0.5 × 10^9^/L. In concordance with previous study [3], we also found that patients with ALC-15 ≥ 0.5 × 10^9^/L had a clear PFS benefit compared to those with lower ALC-15 levels.

The recovery of NK cells and CD3^+^ lymphocytes to pretransplant levels appears about one month after AHCT, whereas the recovery of CD3^+^CD4^+^ cells may take over a year [15]. However, the recovery of functionality in those cells may take years [16]. During the last 20 years, several retrospective [2,17,18,19,20] and few prospective [3,21] studies have demonstrated ALC-15 level ≥ 0.5 × 10^9^/L to be a marker of early immune recovery with survival benefit in NHL patients. However, not all studies have found such a correlation [22,23]. In the most recent study, Porrata et al. showed a predictive role of higher ALC-15 after AHCT for prolonged PFS and OS in patients with aggressive double-hit lymphomas [24]. In concordance with most previous studies, we found in the present study a strong correlation between ALC-15 ≥ 0.5 × 10^9^/L and post-transplant outcome in a patient population including both aggressive and indolent lymphoma types. However, the precise mechanism for the survival benefit of a higher ALC-15 level is still unclear. 

In most publications, the graft CD34^+^ cell count has not been correlated with ALC-15 ≥ 0.5 × 10^9^/L [2], but contradictory findings have also been presented [14,18]. We observed no correlation with infused graft CD34^+^ cell or more primitive CD34^+^CD133^+^CD38^−^ cell counts and ALC-15 ≥ 0.5 × 10^9^/L in multivariate analysis, whereas they were linked with a higher ALC-15 level in the univariate model. CD34^+^ cell count has been for a long time considered the most important graft component regarding hematologic recovery and post-transplant outcome [12], but apparently is less important for early immune recovery after AHCT.

In our study, the mobilization capacity manifested by peak blood CD34^+^ cell count was associated with ALC-15 ≥ 0.5 × 10^9^/L. An explanation for that may possibly be less myelotoxic chemotherapies given previously combined with better preserved immune cells, and this phenomenon *per se* may predict a better outcome. We also found a strong association with graft CD3^+^CD4^+^ cell counts, with a cut-off of 31.8 × 10^6^/kg and ALC-15 ≥ 0.5 × 10^9^/L. In addition, cytotoxic graft CD3^+^CD8^+^ cells > 28.8 × 10^6^/kg were linked with ALC-15 level, corroborating the results in a previous study with a limited number of patients [8]. Also, the infused graft NK cells have been shown to have a role in post-transplant lymphocyte kinetics by predicting a higher ALC-15 level [3,25]. A similar trend was found in our study, including patients, of whom 35% had received plerixafor rescue due to poor mobilization. Surprisingly, the mobilization method used had no effect on early lymphocyte recovery, highlighting the importance of unknown disease and patient-specific factors on early immunologic reconstitution. 

The time frame from the last chemotherapy to the start of apheresis has been shown to have an influence on the pre-apheresis lymphocyte count, which in turn has been suggested to have a prognostic effect [26]. Also, a shorter time from diagnosis to AHCT has been proven to positively influence early immune recovery [27], partly due to fewer pretransplant cytotoxic therapies used and more baseline immune cells that have survived. In line with that, the time from the start of mobilization to AHCT was also correlated with the ALC-15 level in the present study.

The autologous graft-versus-tumor effect has gained attention recently as cumulative data on the effects of autograft cellular composition have been studied [6]. However, to date, the optimal graft composition is still unclear. In the present study, the number of apheresis sessions correlated significantly with the graft lymphocyte subsets in the whole population, whereas in the patients without plerixafor rescue, the correlation was detected only for CD3^+^CD8^+^ cells and NK cells. Of note, all the patients in our study were mobilized with chemotherapy + G-CSF. Previous studies [28] have demonstrated that G-CSF alone mobilization leads to a higher number of lymphocytes in the grafts. Therefore, predictive factors as well as cut-offs for graft cellular composition may differ from those observed in this study in patients mobilized with chemotherapy followed by G-CSF. The immunological status of a given patient may be critical for the mobilization of immune cells in the collected blood grafts. By using mobilization with G-CSF only, making more collections and using PLER if needed, it is possible to increase the lymphocyte content in the grafts to hasten immune recovery and potentially improve outcomes [14]. However, the first study regarding the lymphocyte count modification in autografts has already been published [21,25], albeit more prospective studies are needed aiming to give more lymphocytes after high-dose therapy in NHL patients. 

There are some limitations to this study. Due to the observational nature of the study, variation in the chemotherapies used before and during CD34^+^ cell mobilization was detected. In addition, even though detailed graft cellular composition was available in the great majority of patients, there were some missing data points, which may hamper the results. The strengths of our prospective study encompass comparable practices in graft collection and centrally analyzed graft cellular content by a single experienced cytometrist, as well as a large number of patients with graft cellular analysis available. 

To conclude, mobilization capacity of CD34^+^ cells and detailed parameters of infused graft cellular content mark are prognostic tools that predict ALC-15 ≥ 0.5 × 10^9^/L. In addition, ALC-15 appears to be a useful surrogate marker for a better post-transplant outcome in NHL patients. Patients not achieving the expected lymphocyte recovery after transplantation might benefit from additional therapies, if available, to avoid relapse or progression.

## Figures and Tables

**Table 1:** Characteristics and demographics of 178 patients with non-Hodgkin lymphoma.

Variable	n (%)
**Age, years (median, range)**	61 (19–73)
**Gender**	
** Female**	73 (41)
** Male**	105 (59)
**Histology**	
** DLBCL**	93 (52)
** MCL**	45 (25)
** PTCL**	27 (15)
** FL**	13 (8)
**BM infiltration at diagnosis**	63 (36)
**BM infiltration at mobilization of blood grafts**	3 (2)
**Treatment before mobilization ^1^**	
** CHOP/CHOEP/CEOP**	93 (52)
** Nordic MCL protocol**	44 (24)
** DHAP/MINE**	15 (8)
** BBBD**	14 (8)
** Other**	16 (8)
**Mobilization chemotherapy**	
** HD-AraC**	82 (46)
** DHAP**	49 (28)
** ICE**	14 (8)
** BBBD**	12 (7)
** CHOP**	6 (3)
** Other**	15 (8)
**G-CSF used in mobilization**	
** FIL**	49 (27)
** PEG**	85 (47)
** LIPEG**	42 (23)
**PLER use ^2^**	62 (35)
**Disease status pre-AHCT ^3^**	
** CR I**	93 (53)
** PR I**	39 (22)
** CR II**	21 (12)
** PR II**	14 (8)
** Other**	10 (5)
**High-dose therapy**	
** BEAM**	152 (85)
** BEAC**	7 (4)
** Carmustine-thiotepa**	19 (11)

Abbreviations: BBBD = blood–brain barrier disruption including therapy with intra-arterial mannitol infusion followed by methotrexate and carboplatin plus intravenous rituximab, etoposide and cyclophosphamide; BEAM = carmustine–etoposide–cytarabine–melphalan; BEAC = carmustine–etoposide–cytarabine–cyclophosphamide; CHOP = cyclophosphamide–vincristine–doxorubicin–prednisolone; CR = complete remission; DHAP = dexamethasone–cytarabine–cisplatin; FIL = filgrastim; G-CSF = granulocyte colony-stimulating factor; HD-AraC = high-dose cytarabine; LIPEG = lipegfilgrastim; PEG = pegfilgrastim; PLER = plerixafor; PR = partial remission; pre-AHCT = before autologous hematopoietic stem cell transplantation. ^1^ includes: ICE = ifosfamidi–carboplatin–etoposide, EPOCH = etoposide–prednisone–vincristine–cyclophosphamide–doxorubicin), Nordic CNS lymphoma protocol treatment, BFM protocol treatment; ^2^ missing data in 14 patients; ^3^ missing data in 10 patients.

**Table 2:** Collection data of the 178 patients with NHL.

Variable	Median (Range)
**WBC at the time of first apheresis × 10^9^/L**	11.8 (0.9–116)
**Blood CD34^+^ cells × 10^6^/L at the time of first apheresis**	30 (5–538)
**Peak blood CD34^+^ cell count × 10^6^/L**	38 (6–538)
**CD34^+^ cell yield × 10^6^/kg with first apheresis**	2.3 (0.1–25.5)
**Total yield of CD34^+^ cells × 10^6^/kg collected**	3.6 (1.6–25.5)
**Number of aphereses, n (%)** † **1** **2** **3** **4**	88 (50) 61 (34) 22 (12) 7 (4)

† Missing data in four patients. Abbreviations: WBC = white blood cell.

**Table 3:** Cut-off points of graft cellular components associating with ALC-15 ≥ 0.5 × 10^9^/L according to ROC analysis.

Variable	AUC	*p*-Value	Cut-Off	Sensitivity	Specificity
**Number of CD34^+^ cells** **w/a 7-AAD** **(×10^6^/kg)**	0.561	0.273	4.25	0.367	0.776
**Number of CD34^+^ cells** **w 7-AAD** **(×10^6^/kg)**	0.548	0.384	3.25	0.417	0.775
**Number of CD34^+^CD133^+^CD38^−^ cells** **(×10^6^/kg)**	0.623	**0.022**	0.09	0.265	0.735
**Number of CD3^+^ cells** **(×10^6^/kg)**	0.706	**<0.001**	42.8	0.900	0.469
**Number of CD3^+^CD4^+^ cells** **(×10^6^/kg)**	0.730	**<0.001**	31.8	0.817	0.592
**Number of CD3^+^CD8^+^ cells** **(×10^6^/kg)**	0.685	**<0.001**	28.8	0.717	0.612
**Number of CD19^+^ cells** **(×10^6^/kg)**	0.592	0.090	0.019	0.317	0.857
**Number of NK cells** **(×10^6^/kg)**	0.626	**0.020**	4.4	0.733	0.510

Abbreviations: 7-AAD = 7-aminoactinomycin; NK = natural killer; AUC = area under the curve.

**Table 4:** Predictive factors for ALC-15 ≥ 0.5 × 10^9^/L in 178 patients with NHL.

Variable	Univariate Analysis			Multivariate Analysis		
	OR	95% CI	Sig.	OR	95% CI	Sig.
**Gender**						
** Female**	1					
** Male**	0.950	0.477–1.892	0.885			
**Age (years)**						
** <60**	1					
** >60**	1.372	0.685–2.746	0.372			
**Histology**						
** DLBCL**	1					
** MCL**	0.337	0.81–1.403	0.135			
** FL**	0.729	0.320–1.661	0.451			
** PTCL**	1.237	0.434–3.532	0.690			
** Other**	0.0	0	0.999			
**Mobilization chemotherapy used**						
** HD-AraC**	1					
** DHAP**	1.351	0.590–3.092	0.477			
** ICE**	1.618	0.459–5.708	0.454			
** BBBD**	0	0	0.999			
** Other**	2.810	0.697–11.320	0.146			
**G-CSF in mobilization**						
** FIL**	1					
** PEG**	1.855	0.684–5.028	0.225			
** LIPEG**	1.315	0.421–4.104	0.637			
**PLER use**						
** No**	1					
** Yes**	1.633	0.765–3.486	0.205			
**Disease status pre-AHCT**						
** I CR**	1					
** I PR**	0.572	0.241–1.354	0.204			
** II CR**	0.448	0.170–1.400	0.182			
** II PR**	0.381	0.112–1.295	0.122			
** PD**	0.244	0.044–1.353	0.107			
**Collection parameters**						
** Total CD34^+^ cell yield > 4.25 × 10^6^/kg**	2.091	0.896–4.880	0.088	1.160	0.242–5.566	0.853
** Peak blood CD34^+^ number > 45 × 10^9^/L**	1.654	0.832–3.287	0.151	5.157	1.301–20.444	**0.020**
** Number of apheresis**	1.186	0.551–2.553	0.662			
**Graft components (×10^6^/kg)**						
** CD34^+^ cells in the graft with 7-AAD > 3.25**	2.290	1.003–5.229	**0.049**	1.101	0.260–4.666	0.897
** CD34^+^CD133^+^CD38^−^ cells > 0.09**	2.680	1.194–6.017	**0.017**	1.615	0.566–4.612	0.371
** CD3^+^cells > 42.8**	7.962	2.891–21.925	**<0.001**	5.429	0.898–32.817	0.065
** CD3^+^CD4^+^ cells > 31.8**	6.459	2.713–15.375	**<0.001**	2.462	0.599–10.111	0.211
** CD3^+^CD8^+^ cells > 28.8**	3.994	1.788–8.919	**<0.001**	1.751	0.427–7.179	0.437
** NK cells > 4.4**	2.635	1.192–5.824	**0.017**	1.203	0.384–3.767	0.750

Abbreviations: DLBCL = diffuse large B-cell lymphoma; MCL = mantle cell lymphoma; FL = follicular lymphoma; PTCL = peripheral T-cell lymphoma; HD-AraC = high-dose sytarabine; DHAP = dexamethasone–high-dose cytarabine–cisplatin; ICE = ifosfamide–carboplatin–etoposide; BBBD = blood–brain barrier disruption therapy; G-CSF = granulocyte colony-stimulating factor; FIL = filgrastim; PEG = pegfilgrastim; LIPEG = lipegfilgrastim; PLER = plerixafor; CR = complete remission; PR = partial remission; PD = progressive disease; 7-AAD = 7-amino actinomycin D.

## Data Availability

The datasets created and analyzed during the study are available from the corresponding author on reasonable request.
